# Supplementary material for: Epistasis mediates the evolution of the receptor binding mode in recent human H3N2 hemagglutinin
Source: Nat Commun. 2024 Jun 18;15:5175. doi: 10.1038/s41467-024-49487-4 (PMC11189414; doi:10.1038/s41467-024-49487-4)
Supplement: Supplementary file 1 — Supplementary Information [file 41467_2024_49487_MOESM1_ESM.pdf]

Supplementary Table 1. X-ray data collection and refinement statistics.

	Vic20 HA	Vic20 HA + LSTc
**PDB**	8FAQ	8FAW
**Data collection**		
Wavelength (Å)	1.12723	0.97872
Resolution (Å)	2.04	2.16
Resolution Range [a]	42.92 - 2.04 (2.11 - 2.04)	58.50 - 2.16 (2.24 - 2.16)
Space group	H 3 2	H 3 2
Cell dimensions		
a, *b*, *c* (Å)	99.71, 99.71, 395.31	100.35, 100.35, 395.50
α, *β*, *γ* (°)	90.00, 90.00, 120.00	90.00, 90.00, 120.00
Total reflections	96,219	79,967
Unique reflections	48,124	39,995
Multiplicity [a]	2.0 (2.0)	2.0 (2.0)
Completeness (%) [a]	98.6 (98.1)	96.0 (90.6)
$<I/\sigma_I>$ [a]	22.4 (2.6)	27.6 (2.5)
R_{merge} [a]	0.01 (0.24)	0.01 (0.26)
R_{meas} [a]	0.02 (0.34)	0.02 (0.37)
$CC_{1/2}$ [a]	1.00 (0.91)	1.00 (0.90)
**Refinement**		
Resolution (Å)	42.92 - 2.04	58.50 - 2.16
No. reflections	46,046	38,014
R_{work}^{c} / R_{free}^{d}	0.204/.229	0.202/0.237
No. atoms		
Protein	3,864	3,925
Sugar/Ligand	84	84
Water	141	119
B-factors		
Protein	54.5	51.3
Sugar/Ligand	77.2	80.8
Water	55.4	51.2
RMSD from ideal geometry		
Bond lengths (Å)	0.008	0.008
Bond angles (°)	1.588	1.497

[a] Numbers in parentheses refer to the highest resolution shell.

[b] $R_{merge} = \Sigma |I_i - <I_i>| / \Sigma I_i$ where I_i = the intensity of the ith reflection and $<I_i>$ = mean intensity.

[c] $R_{work} = \Sigma |F_o - F_c| / \Sigma |F_o|$, where F_o and F_c are the observed and calculated structure factors, respectively.

[d] R_{free} was calculated as for R_{work}, but on a test set comprising 5% of the data excluded from refinement.

Supplementary Table 2. Sequence differences between Vic20 HA and Italy20 HA

Residue [a]	Vic20	Italy20
50	Glu	Lys
130	Val	Ile
325	Glu	Asp

[a] H3 numbering. Only residues with different amino acid sequences between Vic20 HA and Italy20 HA are shown.

Supplementary Table 3. Primers for constructing the combinatorial mutant library

Primer ID	Randomized residues (H3 numbering)	Sequence (5' to 3')[a]
#1-F	128, 130, 135, 138	5'-CGT ACG TCT CAT TGG RCT GGA RTC ACT CAA AAC GGA AMA AGT TCT KCT TGC ATA AGG GGT-3'
#1-R	160	5'-TTG GCA TAG TCA CAT TCA GTG CTG GAT ATK TGT AGT TTA AGT GGG TCA ACC-3'
#2-F	None	5'-TAT CCA GCA CTG AAT GTG ACT AT-3'
#2-R	186, 190, 193, 198	5'-TGA TGR TTG AGC ATA CGG GRA GAT TTG GTY CTT GTC CGT AYC CGG GTG GTG AAC CCC CCA-3'
#3-F	193, 198	5'-CCA AAT CTY CCC GTA TGC TCA AYC ATC AGG AAG AAT CAC AGT ATC-3'
#3-R	225	5'-CGT ACG TCT CAT GCT TAT TCT GCT AGG GAT AYC CCT TAT TCT GGG TCT AGA TCC-3'

[a] The symbols for degenerate nucleotides follow the IUPAC nomenclature[1]. R = A or G, M = A or C, K = G or T, and Y = C or T.

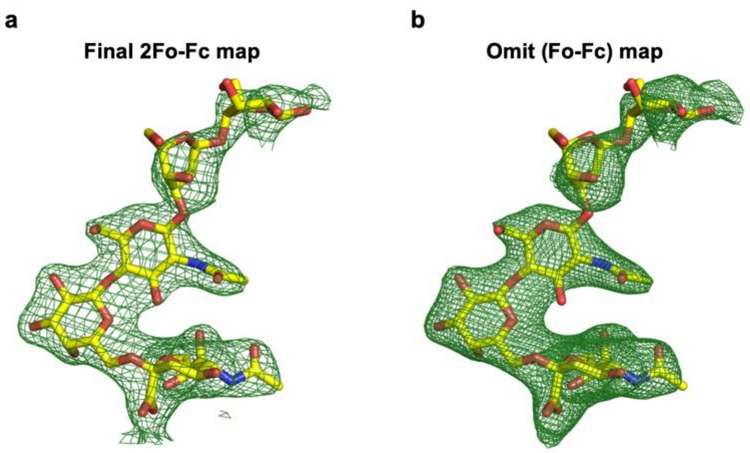

Supplementary Figure 1. Electron density maps for human-type receptor analog LSTc. (a) The final 2Fo-Fc electron density map for LSTc is contoured at 0.8 σ. **(b)** The omit (Fo-Fc) electron density maps for LSTc is contoured at 2.0 σ.

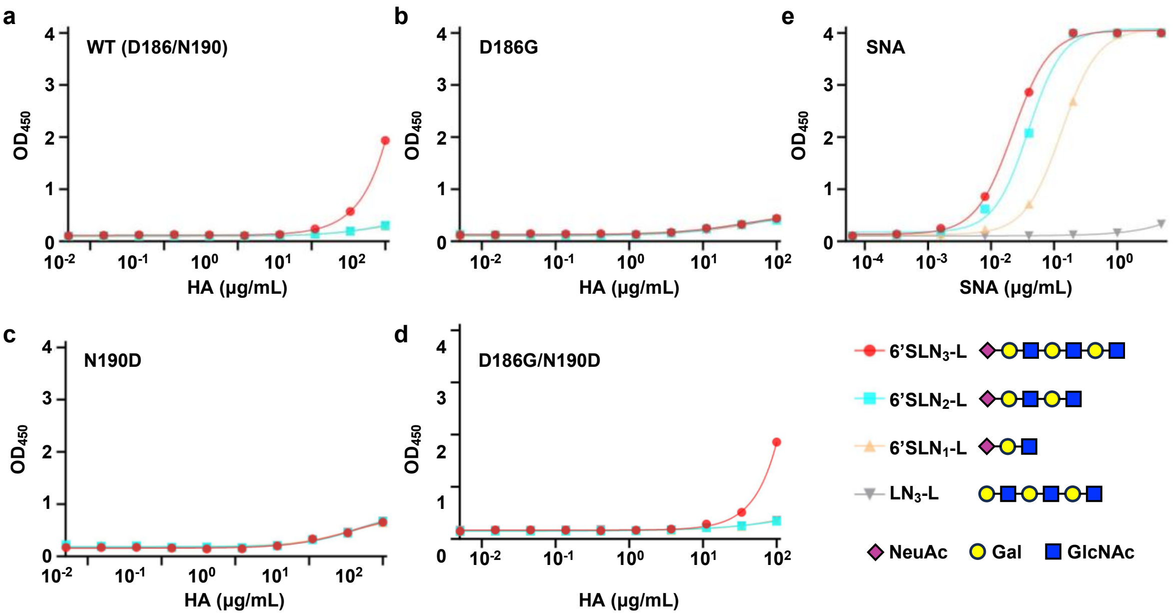

Supplementary Figure 2. Weak avidities of Vic20 HA WT and mutants to linear glycans. (a-e) Binding avidities of **(a-d)** recombinant Vic20 HAs (WT and mutants) and **(e)** sambucus nigra agglutinin (SNA, positive control) to the indicated glycans were measured by ELISA. LN_3-L was used as a negative control. The means of optical density 450 nm (OD_{450}) from three independent experiments are shown with SD indicated by the error bars. -L: linear. Glycan diagrams are drawn according to the Symbol Nomenclature for Glycans recommended by the National Library of Medicine (NLM)[2,3].

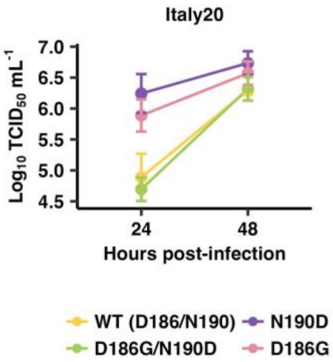

Supplementary Figure 3. Viral replication kinetics of Italy20 WT and mutants. Viral replication kinetics of Italy20 WT and different mutants were compared by infecting hMDCK cells at a multiplicity of infection of 0.001. Viral titers at 24 h and 48 h post-infection were measured by $TCID_{50}$ assay using hMDCK cells. Results are shown as mean titers from three independent experiments. Error bars represent the SD.

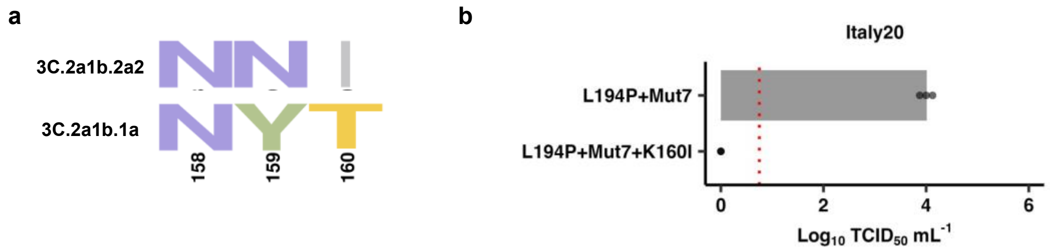

Supplementary Figure 4. Amino acid sequence at residue 160 determines L194P compatibility. (a) The amino acid sequences of HA residues 158-160 of human H3N2 clades 3C.2a1b.1a and 3C.2a1b.2a2 are shown as sequence logos. **(b)** Replication fitness of Italy20 with different combinations of mutations was examined by virus rescue experiments. "Mut7" represents a combination of seven mutations (A128T, I130V, K135T, T160K, D186G, N190D, and S193F). "Mut7+K160I" means that T160K is replaced by T160I in Mut7. Viral titers were measured by $TCID_{50}$. Each data point represents the viral titer of an independent replicate (n = 3). The mean is represented by the bar. The dashed red line represents the lower detection limit.

SUPPLEMENTARY REFERENCES

1 Favre, H. A. & Powell, W. H. *Nomenclature of Organic Chemistry*. DOI: 10.1039/9781849733069 (2013).

2 Neelamegham, S. *et al.* Updates to the symbol nomenclature for glycans guidelines. *Glycobiology* **29**, 620-624 (2019).

3 Varki, A. *et al.* Symbol nomenclature for graphical representations of glycans. *Glycobiology* **25**, 1323-1324 (2015).
