# Supplementary material for: Epistasis mediates the evolution of the receptor binding mode in recent human H3N2 hemagglutinin
Source: Nat Commun. 2024 Jun 18;15:5175. doi: 10.1038/s41467-024-49487-4 (PMC11189414; doi:10.1038/s41467-024-49487-4)
Supplement: Supplementary file 3 — Description of Additional Supplementary Files [file 41467_2024_49487_MOESM3_ESM.pdf]

Description of Additional Supplementary Files

Supplementary Data Legend:

File Name: Supplementary Data 1
Description: Glycan array compound list. Glycan diagrams are drawn according to the Symbol Nomenclature for Glycans recommended.
