# Supplementary material for: Epistasis mediates the evolution of the receptor binding mode in recent human H3N2 hemagglutinin
Source: Nat Commun. 2024 Jun 18;15:5175. doi: 10.1038/s41467-024-49487-4 (PMC11189414; doi:10.1038/s41467-024-49487-4)
Supplement: Supplementary file 5 — Reporting Summary [file 41467_2024_49487_MOESM5_ESM.pdf]

nature portfolio

Corresponding author(s): Chris Mok, Nicholas Wu

Last updated by author(s): May 3, 2024

Reporting Summary

Nature Portfolio wishes to improve the reproducibility of the work that we publish. This form provides structure for consistency and transparency in reporting. For further information on Nature Portfolio policies, see our Editorial Policies and the Editorial Policy Checklist.

Statistics

For all statistical analyses, confirm that the following items are present in the figure legend, table legend, main text, or Methods section.

n/a	Confirmed	
☐	☒	The exact sample size (*n*) for each experimental group/condition, given as a discrete number and unit of measurement
☐	☒	A statement on whether measurements were taken from distinct samples or whether the same sample was measured repeatedly
☐	☒	The statistical test(s) used AND whether they are one- or two-sided<br>*Only common tests should be described solely by name; describe more complex techniques in the Methods section.*
☒	☐	A description of all covariates tested
☒	☐	A description of any assumptions or corrections, such as tests of normality and adjustment for multiple comparisons
☐	☒	A full description of the statistical parameters including central tendency (e.g. means) or other basic estimates (e.g. regression coefficient) AND variation (e.g. standard deviation) or associated estimates of uncertainty (e.g. confidence intervals)
☐	☒	For null hypothesis testing, the test statistic (e.g. *F*, *t*, *r*) with confidence intervals, effect sizes, degrees of freedom and *P* value noted<br>*Give P values as exact values whenever suitable.*
☒	☐	For Bayesian analysis, information on the choice of priors and Markov chain Monte Carlo settings
☒	☐	For hierarchical and complex designs, identification of the appropriate level for tests and full reporting of outcomes
☒	☐	Estimates of effect sizes (e.g. Cohen's *d*, Pearson's *r*), indicating how they were calculated

Our web collection on statistics for biologists contains articles on many of the points above.

Software and code

Policy information about availability of computer code

Data collection	Sequences used were downloaded from GISAID (http://gisaid.org). Protein structure data were collected at the Advanced Photon Source.
Data analysis	Sequences were aligned using MAFFT v7 and selected for analysis using CD-HIT. Phylogenetic tree was built using IQ-TREE2 and visualized by ggtree package in R. Next-generation sequencing data were obtained in FASTQ format. Forward and reverse reads of each paired-end read were merged by PEAR. The merged reads were parsed by SeqIO module in BioPython. Initial diffraction data of crystallized protein were indexed, integrated, and scaled using autoPROC. The structure was solved by molecular replacement using Phaser-MR included in the Phenix suite, refined using REFMAC5, and manually built in COOT. Ramachandran statistics were calculated using MolProbity. Custom codes to process next-generation sequencing data can be accessed at: https://doi.org/10.5281/zenodo.11099315.

For manuscripts utilizing custom algorithms or software that are central to the research but not yet described in published literature, software must be made available to editors and reviewers. We strongly encourage code deposition in a community repository (e.g. GitHub). See the Nature Portfolio guidelines for submitting code & software for further information.

Data

Policy information about availability of data

All manuscripts must include a data availability statement. This statement should provide the following information, where applicable:

- Accession codes, unique identifiers, or web links for publicly available datasets
- A description of any restrictions on data availability
- For clinical datasets or third party data, please ensure that the statement adheres to our policy

Raw sequencing data have been deposited to the NIH Short Read Archive under accession number: PRJNA883249. The X-ray coordinates and structure factors have been deposited in the RCSB Protein Data Bank under accession codes 8FAQ and 8FAW.

Research involving human participants, their data, or biological material

Policy information about studies with human participants or human data. See also policy information about sex, gender (identity/presentation), and sexual orientation and race, ethnicity and racism.

Reporting on sex and gender	Sex or gender information of human participants were provided by self-reporting. The information of sex or gender was not analyzed in this study because it was not a major factor affecting the results.
Reporting on race, ethnicity, or other socially relevant groupings	No information of race, ethnicity or other socially relevant groupings were used for this study.
Population characteristics	Participants involved in this study were all healthy adults with past infection or vaccination history of human H3N2 virus.
Recruitment	Human plasma samples from 479 healthy adults in Hong Kong in 2021 and 2022 were obtained from previous serological study. We selected 28 plasma samples for the present study based on two criteria: 1) the donors were not infected or vaccinated with recent H3N2 virus with HA D186/N190, and 2) plasma samples had 50% microneutralization titer of at least 1:40 against A/Singapore/INFIMH-16-0019/2016.
Ethics oversight	the Joint Chinese University of Hong Kong-New Territories East Cluster (Ref no: 2020.229) Clinical Research Ethics Committee.

Note that full information on the approval of the study protocol must also be provided in the manuscript.

Field-specific reporting

Please select the one below that is the best fit for your research. If you are not sure, read the appropriate sections before making your selection.

☐ Life sciences ☐ Behavioural & social sciences ☒ Ecological, evolutionary & environmental sciences

For a reference copy of the document with all sections, see nature.com/documents/nr-reporting-summary-flat.pdf

Ecological, evolutionary & environmental sciences study design

All studies must disclose on these points even when the disclosure is negative.

Study description	This study describes the evolution of hemagglutinin protein in recent human H3N2 virus, and elucidates the mechanism in terms of protein structure, protein stability, receptor binding mode mediated by epistasis on the receptor-binding site.
Research sample	Serum samples from female BALB/c mice aged at 6-8 weeks, and plasma samples from 28 healthy human adults were used in this study. Human samples were originally collected for a previous study (PMID: 37481108).
Sampling strategy	Six mice were used in this study as it was sufficient for statistical analysis (more than three biological replicates are required). Since human immune response is more variable, we selected 28 from 479 human plasma samples that met the criteria, which could be representative and sufficient for the analysis.
Data collection	All data were recorded in excel file or formatted in PDB, fasta or fastq, aln files. Data were collected, processed and analyzed by all co-authors.
Timing and spatial scale	Data were collected between January 2021 and March 2024.
Data exclusions	No data was excluded.
Reproducibility	Experiments were performed in triplicate to ensure the reproducibility of results and conclusions.
Randomization	Data obtained from six mice are all included in this study without randomization. For human plasma samples, 28 out of 479 samples

Randomization	were selected based on their high level of neutralizing titers against older human H3N2 strain. This would not affect the results or conclusions because these were used as seropositive samples to human H3N2 virus and for calculating the decreased fold-change against recent human H3N2 strains.
Blinding	Blinding was applicable in this study since all data are objective.

Did the study involve field work? ☐ Yes ☒ No

Reporting for specific materials, systems and methods

We require information from authors about some types of materials, experimental systems and methods used in many studies. Here, indicate whether each material, system or method listed is relevant to your study. If you are not sure if a list item applies to your research, read the appropriate section before selecting a response.

Materials & experimental systems

n/a	Involved in the study
☐	☒ Antibodies
☐	☒ Eukaryotic cell lines
☒	☐ Palaeontology and archaeology
☐	☒ Animals and other organisms
☒	☐ Clinical data
☒	☐ Dual use research of concern
☒	☐ Plants

Methods

n/a	Involved in the study
☒	☐ ChIP-seq
☒	☐ Flow cytometry
☒	☐ MRI-based neuroimaging

Antibodies

Antibodies used	anti-His mouse IgG2a antibody (Biolegend, #362616), HRP-conjugated goat anti-mouse IgG (H+L) secondary antibody (Invitrogen, #G21040), and Alexa488-conjugated anti-mouse antibody (Invitrogen, #A28175)
Validation	No primary antibody is used in this study. All secondary antibodies used are anti-mouse and validated to be used in western blot, immunocytochemistry and flow cytometry staining. More information can be found on Biolegend and Invitrogen websites with the category numbers above.

Eukaryotic cell lines

Policy information about cell lines and Sex and Gender in Research

Cell line source(s)	Human embryonic kidney 293T cells (ATCC), humanized Madin-Darby canine kidney (hMDCK) cells (from Yoshihiro Kawaoka)34, MDCK-SIAT1 cells (ATCC), Sf9 cells were used in this study.
Authentication	All cell lines used were authenticated.
Mycoplasma contamination	All cell lines were tested negative for mycoplasma contamination.
Commonly misidentified lines (See ICLAC register)	No commonly misidentified cell lines was used in this study.

Animals and other research organisms

Policy information about studies involving animals; ARRIVE guidelines recommended for reporting animal research, and Sex and Gender in Research

Laboratory animals	Female BALB/c mouse aged at 6-8 weeks
Wild animals	This study does not involve any wild animals.
Reporting on sex	Six female BALB/c mice were used in this study. We chose female mice for our experiment as they are less aggressive and variable in humoral immune response compared to male BALB/c. While our study aimed to compare the neutralizing capacity of mice sera against different human H3N2 mutants, the gender of the animal would not be a major factor influencing the results or conclusions.
Field-collected samples	Mice were kept at 18-22 degrees with 40-60% humidity. Housing condition was 12h light/12h dark cycle.
Ethics oversight	All animal procedures were carried out in accordance with institutionally approved protocols of The University of Hong Kong (Approval number: 5598-20).

Note that full information on the approval of the study protocol must also be provided in the manuscript.

Plants

Seed stocks	*Report on the source of all seed stocks or other plant material used. If applicable, state the seed stock centre and catalogue number. If plant specimens were collected from the field, describe the collection location, date and sampling procedures.*
Novel plant genotypes	*Describe the methods by which all novel plant genotypes were produced. This includes those generated by transgenic approaches, gene editing, chemical/radiation-based mutagenesis and hybridization. For transgenic lines, describe the transformation method, the number of independent lines analyzed and the generation upon which experiments were performed. For gene-edited lines, describe the editor used, the endogenous sequence targeted for editing, the targeting guide RNA sequence (if applicable) and how the editor was applied.*
Authentication	*Describe any authentication procedures for each seed stock used or novel genotype generated. Describe any experiments used to assess the effect of a mutation and, where applicable, how potential secondary effects (e.g. second site T-DNA insertions, mosiacism, off-target gene editing) were examined.*
